# Zero-Dose Children and Nutrition-Service Gaps: A Case for Integrated Delivery in Low- and Middle-Income Countries

**DOI:** 10.3390/vaccines14090740

**Published:** 2026-08-27

**Authors:** Kevin Tang, Uchenna Agu, Fanny Sandalinas, Andreas Hasman, Annette Imohe, Ibrahim Dadari

**Affiliations:** 1 Department of Population Health, London School of Hygiene & Tropical Medicine, London WC1E 7HT, UKuchenna.agu1@alumni.lshtm.ac.uk (U.A.);; 2 United Nations Children’s Fund (UNICEF), New York, NY 10017, USA; 3 United Nations Children’s Fund (UNICEF)—Ghana Country Office, Accra P.O. Box 5051, Ghana

**Keywords:** zero-dose, nutrition, immunization, integrated service delivery

## Abstract

**Background/Objectives:** Zero-dose children, defined as those who have not received any routine vaccines, are a central priority of global immunization equity efforts and are widely considered to be among the most deprived children. This study examined whether zero-dose status also identifies children who have not received nutrition services and who are nutritionally vulnerable. **Methods:** We analyzed data from the Demographic and Health Surveys and Multiple Indicator Cluster Surveys across 55 low- and middle-income countries, including 560,314 children aged 12 to 23 months. We assessed the association between zero-dose status and non-receipt of vitamin A supplementation (VAS) and deworming, and between zero-dose status and the Multidimensional Poverty Index (MPI). **Results:** Zero-dose status was weakly associated with non-receipt of VAS and deworming (most phi coefficients between 0.1 and 0.3) and with the MPI (mean 0.11), and showed negligible correlation with child nutritional status, measured by stunting and wasting (mean phi 0.03). Most zero-dose children nonetheless had not received these nutrition services, with 62% not receiving VAS and 72% not receiving deworming, amounting to an estimated 12.5 and 14.4 million children, concentrated in a small number of countries. This reflected the generally low coverage of nutrition interventions across all children rather than a specific concentration among the zero-dose. **Conclusions:** Immunization outreach platforms can therefore be used to extend nutrition services to large numbers of under-reached children.

## 1. Introduction

Child health and nutrition are interdependent [1], and a comprehensive package of health and nutrition interventions provided to all children underpins the global goal of universal health coverage [2]. Immunization has been estimated to have averted more than 154 million deaths since the establishment of the Expanded Programme on Immunization (EPI), the majority among children under five years of age [3,4], and a core package of 22 evidence-backed maternal and child nutrition interventions has been estimated to reduce under-five mortality by approximately 15% [5]. Both programmes nonetheless encounter a common difficulty, namely the identification of the most vulnerable children and the delivery of services to them. Children in the poorest and most marginalized households experience the greatest burden of morbidity and mortality [6,7], and these risks are frequently compounded by co-deprivations that simultaneously increase the need for intervention and reduce access to it [8].

Within the immunization field, this difficulty has increasingly been framed in terms of “zero-dose” children, a widely used equity indicator in global immunization programmes and the Immunization Agenda 2030. Zero-dose children are defined as those who have not received any vaccinations and are, in practice, measured by the absence of the first dose of a diphtheria-tetanus-pertussis-containing vaccine (DTP1) [9]. Zero-dose children are generally among the most deprived: they are disproportionately found in the poorest households, in remote rural areas, urban informal settlements, and conflict-affected settings, and their mothers tend to have lower levels of education and autonomy [10,11,12]. The reduction in zero-dose numbers has become a prominent and well-resourced objective across several global frameworks, including the Immunization Agenda 2030 [13], the UNICEF Immunization Roadmap 2018–2030 [14], and the Gavi Alliance 5.0 Strategy 2021–2025 [15]. An estimated 14.5 million zero-dose children remain worldwide [16,17], and considerable operational investment, including equity-focused financing, has been directed towards identifying and vaccinating them. In addition, following the substantial backsliding from the COVID-19 pandemic, which has pushed the immunization program to be off its zero-dose targets of the IA2030, the Big Catch-Up initiative was designed to recover the accumulated zero-dose and under vaccinated children from the pandemic years, while strengthening the health system for accelerated progress [18].

The concentration of resources on a clearly defined group of highly excluded children raises the question of whether the same children are also those most frequently missed by other essential services, including nutrition interventions, and whether the platforms developed to reach them might be used to deliver such services. There are reasons to expect that these populations will overlap: the economic, geographic, and social barriers that leave children unvaccinated also impede access to nutrition services, and children missed by high-dose vitamin A supplementation (VAS), for example, tend to face the same constraints [19]. The case for joint delivery is further supported by the biological relationship between the two domains, as malnutrition and infection are mutually reinforcing [20,21]: malnutrition weakens immune function and exacerbates infection, while infection depletes nutritional status by increasing metabolic demand, impairing nutrient absorption, and inducing nutrient loss [22]. Soil-transmitted helminth infections remain a major public health concern in many low- and middle-income countries. These infections can impair nutritional status through multiple pathways, including reducing nutrient absorption, intestinal blood loss, contributing to anemia. As a result, the WHO recommends periodic preventive chemotherapy (deworming) for children living in endemic areas. VAS and deworming are, moreover, delivered to children of overlapping ages and have logistical requirements similar to those of immunization [23], and their integration with immunization services has been proposed as a means of improving the coverage and efficiency of both.

Immunization programmes targeting zero-dose children already employ a range of pro-equity delivery approaches, including adjusted immunization schedules, community engagement, and specialized outreach in the settings where these children are most concentrated [12,24,25]. Because zero-dose children are, by definition, not reached through routine immunization contacts, it is these outreach mechanisms, rather than routine service delivery, that would constitute the platform onto which nutrition services could be added. Such functional integration, encompassing coordinated planning, financing, supply chains, and supervision, is consistent with the WHO–UNICEF operational framework for primary health care [26]. Taken together, these considerations form a coherent rationale for using zero-dose immunization platforms to deliver nutrition services.

This rationale rests, however, on an assumption that has not been examined empirically at scale, namely that zero-dose status identifies children who have lower access to nutrition services or who are nutritionally vulnerable. The distinction is consequential for the way in which integration is justified. If zero-dose status were strongly associated with nutrition-service access and nutritional vulnerability, it could function as a practical entry point for targeting nutrition interventions. If the association were weak, the rationale for co-delivery would rest instead on shared delivery infrastructure and on the absolute number of zero-dose children not receiving nutrition services who could be reached, rather than on the precision with which zero-dose status identifies nutritional need. Establishing which of these applies is relevant to the design of integrated programmes intended to be both efficient and equitable.

This study aims to establish, at scale, whether immunization platforms targeting zero-dose children represent a viable route for extending essential nutrition services to underserved children, and on what basis such integration can be justified. Using nationally representative survey data from 55 low- and middle-income countries, this study addresses two questions. First, it examines the extent to which zero-dose children are also not receiving VAS and deworming and estimates the number of children affected. Second, it assesses how strongly zero-dose status is associated with non-receipt of nutrition services and with broader nutritional and multidimensional vulnerability, in order to evaluate its utility as an indicator for nutrition targeting.

## 2. Materials and Methods

### 2.1. Data Sources and Descriptions

This is a secondary analysis using data from the Demographic and Health Surveys (DHSs) and the Multiple Indicator Cluster Surveys (MICSs). Both survey systems aim to generate a wide range of standardized data on core indicators characterizing population development and well-being across multiple countries [27]. Sampling protocol deploys a two-stage sampling procedure, where first, enumeration areas are defined geographically into primary sampling units; then, systematic sampling identifies ~20 households for inclusion. Selected households are visited by trained interviewers who administer the questionnaire to key members of the household.

We extracted data from DHS’s Woman’s Questionnaire and the MICS’ Infants and Children Questionnaire, both containing information on children born to the respondent. The questionnaires are designed to capture retrospective data, relying on a women’s birth recall and, or evidence from their home-based records or vaccination cards, which includes the timing and details of each live birth, as well as health and nutrition practices. From the selected households, all mothers aged 15–49 years were asked to present vaccination cards of children born in the 5 years before the survey [28]. If no card was presented, the interviewer asked the mother to recall all vaccinations, and if appropriate, the number of doses received.

We included countries conducting a DHS or MICS since 2012 and designated a priority for vitamin A supplementation by UNICEF [29] or were eligible for vaccine programme support according to Gavi, the Vaccine Alliance [30]. For countries where multiple surveys have been implemented since 2012, the most recent survey was used to characterize the country. The MICS questionnaire does not include questions about the nutrition interventions of interest to this study, and these countries were therefore included only in the analysis of multidimensional vulnerability and excluded from the analyses of VAS and deworming. We used the UNICEF regional classification system, which groups countries into the following seven regions based on operational and programmatic criteria: East Asia and the Pacific, Europe and Central Asia, East and Southern Africa, Latin America and the Caribbean, the Middle East and North Africa, South Asia, and West and Central Africa.

### 2.2. Primary Indicators: Zero-Dose Status and Nutrition-Intervention Access

We used a proxy indicator to represent the national proportion of zero-dose children consistent with global guidance [13] and the existing literature [9]. For the numerator, we classified children as zero-dose if they had not received their first dose of the diphtheria-tetanus-pertussis-containing vaccine (DTP1) at 12 months of age as per recommended national EPI schedule, which aligns with the immunization schedule for infants recommended by WHO to be given at 6 weeks of life [4]. DTP1 is widely used as the standard indicator of access to routine immunization services and as the operational measure of zero-dose status. We considered any report of DTP1 receipt as a valid indication of vaccination irrespective of the schedule of administration or the presence of documentation [31]. We excluded children under 12 months who had potentially not completed their full vaccination schedule to limit the impact of censored observations on the proportion of zero-dose children.

We represented other child health and nutrition programme intervention access using the following two widely implemented nutrition programmes with direct impact on morbidity and mortality: high-dose vitamin A supplementation (VAS) and prophylaxis of soil-transmitted helminths (deworming). We classified children as non-receipt of VAS interventions if they did not receive any or both of their biannual dose(s) of VAS in the past six months, which aligns with WHO recommendation for infants and children 6–59 months of age to receive a dose of VAS every 4–6 months in contexts where vitamin A deficiency is a public health problem [32]. We classified children as non-receipt of deworming interventions if they did not receive a biannual dose of deworming medication in the past six months, which aligns with WHO recommendation for children 12–59 months to receive preventive chemotherapy, using annual or biannual single dose of albendazole (400 mg) or mebendazole (500 mg) [33]. Non-receipt may reflect a lack of access, but it may also arise from interruptions in supply, the timing of campaigns, or a child’s ineligibility at the time of the survey, and should therefore be interpreted as an indicator of programmatic non-coverage rather than confirmed unmet need. For the purpose of our analysis we referred to these as either “ZD-VAS” for children not receiving vitamin A supplementation and “ZD-Deworming” for children not receiving deworming.

### 2.3. Scale of Non-Receipt Among Zero-Dose Children

We assessed the extent and number of zero-dose children not receiving nutrition interventions in two ways. First, we characterized the potential for nutrition intervention integration by estimating the share of zero-dose children not receiving each nutrition intervention for 12- to 23-month-old children in each country. Countries with higher proportions of zero-dose children not receiving nutrition interventions indicate contexts where integration with co-delivered interventions could have the greatest potential to fulfil previously unmet child health and nutrition needs. Second, we estimated the number of children between 12 and 35 months who were zero-dose and did not receive nutrition interventions using deterministic projection. The age range was expanded to incorporate children up to 35 months to maintain better consistency with the administration schedules for both VAS and deworming [32,33], and DHS standard guidance recommends collecting vaccination histories for all children up to 35 months [34].

The projected population was generated by combining the number of children between 12 and 35 months of age in the country, the proportion of zero-dose children in the country, and the proportion of these children not receiving each nutrition intervention based on the DHS survey. The total number of children in the projected population was obtained from the UN World Population Prospects [35] which provided the number of children for each country age-stratified by one-year birth cohorts. Population denominators were drawn from the World Population Prospects estimate for the same calendar year as each country’s survey. The proportion of zero-dose children was estimated separately for each birth cohort divided into 12-month-age intervals at the time the survey was implemented, or age 12- to 23-month- and 24- to 35-month-old children. The proportion of zero-dose children not receiving each nutrition intervention was estimated from the survey data analysis. The total number of zero-dose children not receiving each nutrition intervention was aggregated for each country, region, and globally for all countries included in this study. This projection yielded a single point estimate for each country and did not incorporate sampling or model uncertainty.

### 2.4. Zero-Dose Status as an Indicator of Nutritional Vulnerability

We examined how strongly zero-dose status was associated with non-receipt of nutrition services and with broader child vulnerability to evaluate its utility as an indicator for targeting. For these analyses we included all surviving children aged 12 to 23 months and estimated the proportion of zero-dose children for the full calendar year in which the survey was implemented. This age range is consistent with national EPI implementation efforts, where most countries aim to have completed their primary immunization schedule by 12 months and limiting respondent bias with a recent and narrow recall window [36].

We assessed the correlation between zero-dose status and non-receipt of VAS or preventive chemotherapy for deworming. Phi correlation coefficients (ϕ) for each country described the association between child zero-dose status and non-receipt of each nutrition intervention. Evaluation of the strength of correlations was consistent with the Cohen thresholds [37], where correlation coefficients <0.1 indicated “no correlation”, ≥0.1 to <0.3 indicated “weak correlation”, ≥0.3 to <0.5 indicated “medium correlation”, and ≥0.5 indicated “strong correlation”.

We assessed the correlation between zero-dose status and an adapted, child-level version of the Global Multidimensional Poverty Index (MPI) [38]. The standard Global MPI is a widely used internationally comparable indicator of acute poverty constructed from ten indicators nested within the following three dimensions: health, education, and living standards. Because our indicator set was limited to variables available for children in the DHS and MICSs, we constructed a simplified version using one indicator per dimension. Nutrition was represented by whether the included children were stunted and/or wasted, defined as having a height-for-age and/or weight-for-height z-score below −2 standard deviations from the WHO Child Growth Standards median [39]. Education was represented by whether the mother of the included children had completed five or less years of schooling. Living standards were represented by whether the households of included children were deprived in at least two of the following six characteristics defining living standards: cooking fuel, sanitation, drinking water, electricity, dwelling type, and asset ownership. Consistent with the standard Global MPI weighting scheme, each dimension was weighted equally at one-third, and because each dimension in our adaptation was represented by a single indicator, that indicator determined the dimension’s full contribution to the composite score. Children with missing data on any of the three indicators were excluded from this analysis (complete case exclusion).

Point-biserial correlation coefficients for each country described the association between child zero-dose status and the child’s MPI measured continuously. We used this continuous composite score rather than the standard binary classification of children, as our objective was to assess the strength of association rather than to classify individual children as poor or non-poor. Furthermore, phi correlation coefficients (ϕ) for each country described the association between child zero-dose status and each of the three individual dimensions of poverty included in the MPI (i.e., nutrition, education, and living standards). Evaluation of the strength of correlations was consistent with the Cohen thresholds [37] as described previously. Because the phi coefficient is sensitive to the marginal distributions of the two binary variables, its maximum attainable value is reduced when the prevalence of either variable is far from 50%, as was the case for zero-dose status in many countries. The coefficients reported here should be interpreted with this property in mind. Correlation coefficients for each country were compared to the national zero-dose prevalence to evaluate the utility of zero-dose as an indicator of child vulnerability in countries with varying proportions of the population who are zero-dose.

### 2.5. Data Extraction and Statistical Analysis

DHS data were accessed through the DHS programme’s application programming interface on 15 August 2024 via the functions available on the *rdhs* package. MICS data were accessed through the UNICEF data portal. All analyses accounted for the complex survey design of DHS and MICSs using the *srvyr* package. Survey design objects incorporated sampling weights, primary sampling units (clusters), and stratification variables provided within each survey dataset. Additional data cleaning and management used a variety of functions from the *tidyverse* package. All estimates were documented following the Guidelines for Accurate and Transparent Health Estimates Reporting [40]. Data analysis was conducted in RStudio (Build 554, the R Foundation for Statistical Computing).

## 3. Results

### 3.1. Survey and Population Characteristics

In total, 55 countries and 560,314 children aged 12 to 23 months were included in the analysis. Of these, 93% (*n* = 51) were represented by data from the DHS and 7% (*n* = 4) by data from the MICS. The largest group of countries came from the West and Central Africa region (38%, *n* = 21), followed by East and Southern Africa (31%, *n* = 17) and East Asia and the Pacific (13%, *n* = 7), using the UNICEF regional classification. Not all countries contributed to every analysis. Five countries lacked data on access to nutrition interventions (Angola, the Central African Republic, Eswatini, Lao People’s Democratic Republic, and São Tomé and Príncipe) and were excluded from the analyses of non-receipt of VAS and deworming. Four countries lacked the variables required to construct the Multidimensional Poverty Index (Afghanistan, Indonesia, the Philippines, and Yemen) and were excluded from the analyses of multidimensional vulnerability. One additional country, Ethiopia, had data on vitamin A supplementation but not deworming and was included only in the VAS-specific analyses. Regional and global totals for each intervention are based on all countries with available data for that intervention.

**Figure 1:**
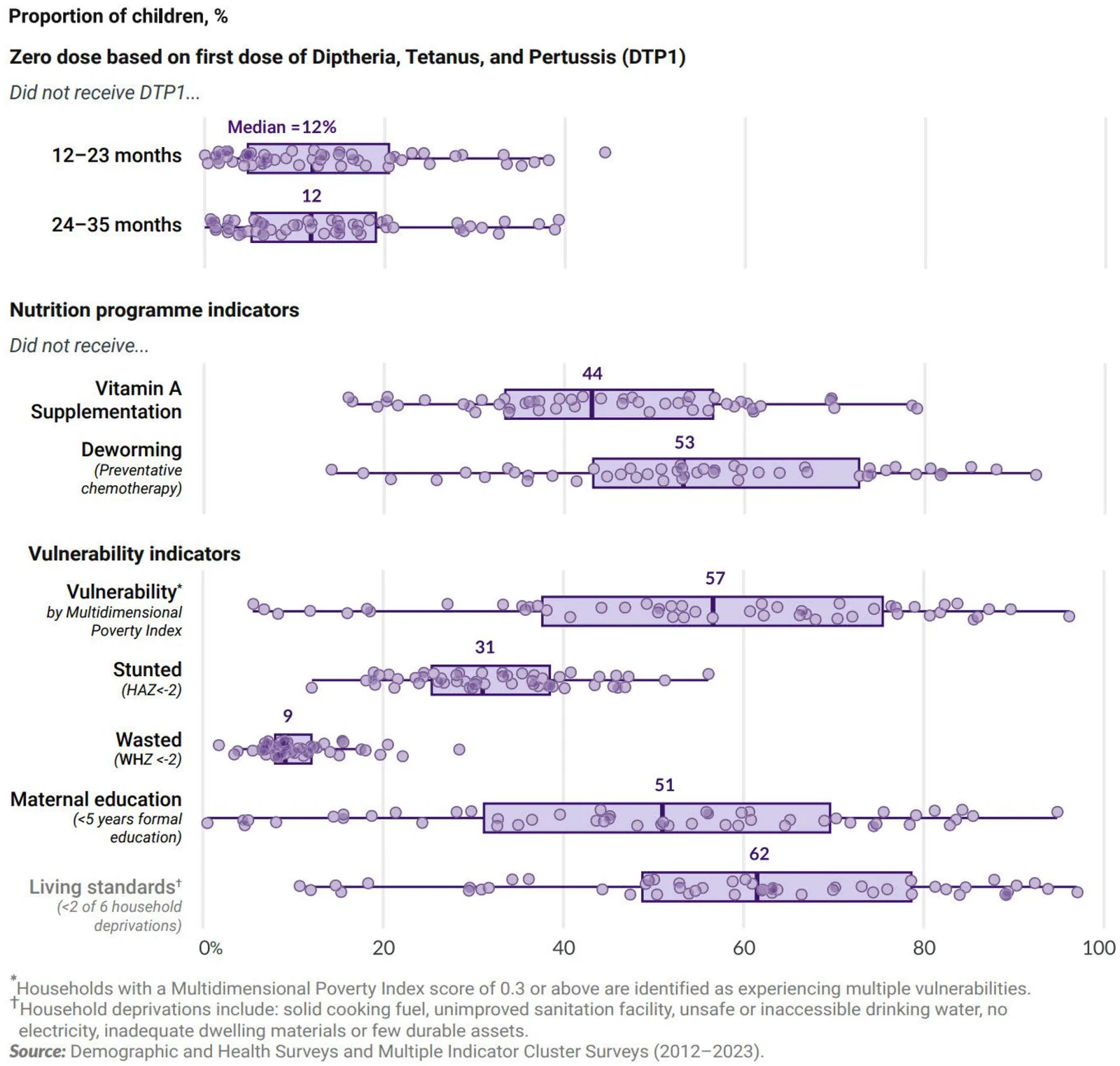
A jittered strip plot with overlayed box plot where the medians and distribution of 10 nutrition and vaccination programme indicators and markers of vulnerability and malnutrition are shown for priority countries. HAZ = height-for-age z-score, WHZ = weight-for-height z-score.

A summary of the zero-dose, nutrition intervention access, and vulnerability indicators by country is presented in Figure 1. The following five countries in West and Central Africa had a zero-dose prevalence exceeding 25%: Chad, the Central African Republic, Guinea, Nigeria, and Côte d’Ivoire. Across all included countries, the median proportion of children not receiving each nutrition intervention was 44% for VAS and 53% for deworming.

### 3.2. Extent and Number of Children Not Receiving Nutrition Interventions

**Figure 2:**
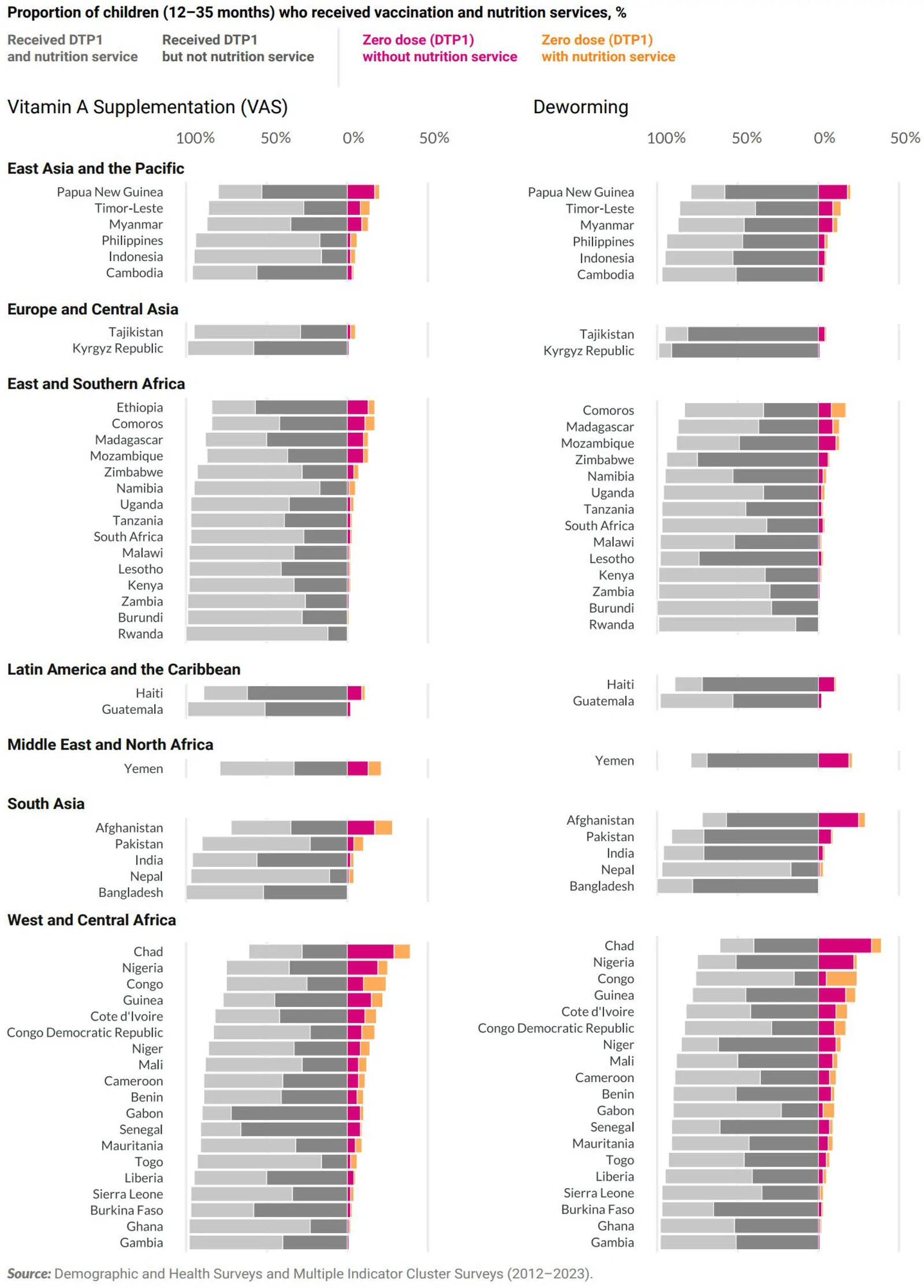
Two horizontal stacked bar charts for (1) vitamin A supplementation (VAS) and (2) deworming treatment, each subset by UNICEF region showing the proportion of children from priority countries in the following four categories: (1) children who received both DTP1 and the nutrition service; (2) children who received DTP1 but not the nutrition service; (3) children who received neither DTP1 or the nutrition service; and (4) children who received the nutrition service but not DTP1.

The mean national prevalence of zero-dose children across surveyed countries was 12%, with wide variation between countries. The ten countries with the highest prevalence were Chad (38%), Guinea (35%), Papua New Guinea (34%), Nigeria (33%), Côte d’Ivoire (28%), Afghanistan (25%), Congo (24%), Ethiopia (23%), Yemen (22%), and Madagascar (21%). The proportion of children who received DTP1 but missed nutrition services and the proportion who were zero-dose and did not receive each nutrition services are presented by country in Figure 2. Across surveyed countries, the mean share of zero-dose children not receiving VAS was 60%, ranging from 16% to 90%. Among the ten highest-prevalence countries, the largest shares not receiving VAS were in Papua New Guinea (82%), Madagascar (80%), Ethiopia (76%), Nigeria (75%), and Chad (73%). The mean share of zero-dose children not receiving deworming was higher, at 73%, ranging from 11% to 100%, with the largest shares in Yemen (92%), Afghanistan (92%), Nigeria (91%), Papua New Guinea (90%), and Chad (83%).

**Figure 3:**
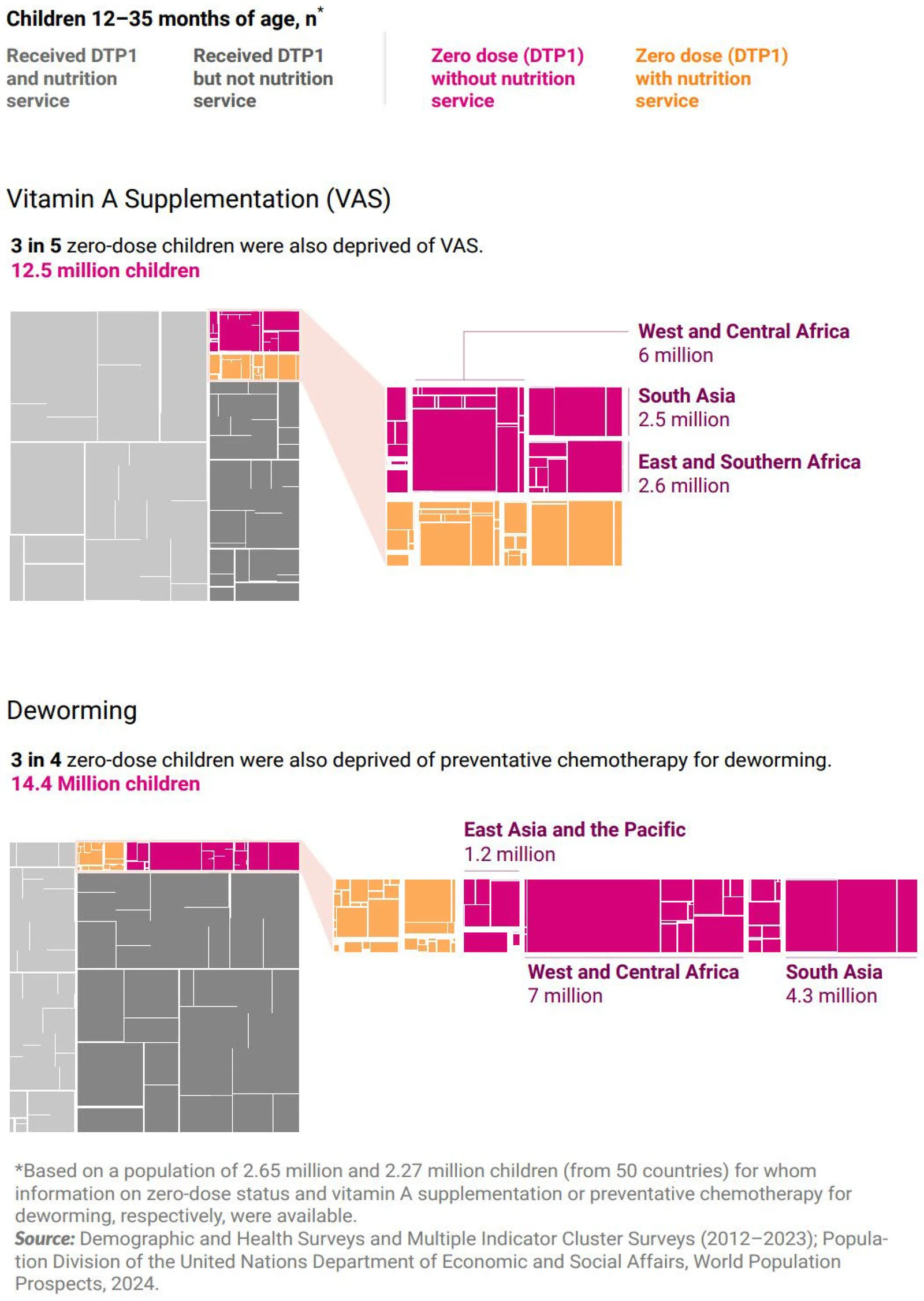
Tree map presenting the projected population of children from priority countries (by country and UNICEF region) in the following four categories: (1) children who received both DTP1 and the nutrition service; (2) children who received DTP1 but not the nutrition service; (3) children who received neither DTP1 or the nutrition service; and (4) children who received the nutrition service but not DTP1.

We then projected these shares onto the underlying child populations to estimate the absolute number of zero-dose children not receiving nutrition services in Figure 3. Of an estimated 149,102,403 children aged 12 to 35 months in the included countries, the projection estimated that 8,649,496 children aged 12 to 23 months and 19,950,611 children aged 12 to 35 months were zero-dose. Of the estimated 19,950,611 zero-dose children aged 12–35 months, approximately 12,457,087 (62%) were classified as not receiving VAS, with the largest numbers in West and Central Africa (6,026,385; a 68% share of the region’s zero-dose children), East and Southern Africa (2,625,717; 74%), and South Asia (2,489,905; 48%). Of the estimated 19,950,611 zero-dose children aged 12–35 months, an estimated 14,403,044 zero-dose children (72%) were classified as not receiving deworming, with the largest numbers in West and Central Africa (7,032,529; 79%), South Asia (4,301,920; 83%), and East Asia and the Pacific (1,285,675; 77%). The country-level means (60% for VAS and 73% for deworming) differ slightly from the projected global estimates (62% and 72%, respectively) because the latter are weighted by the size of the child population in each country.

For both interventions, more than half of all zero-dose children not receiving nutrition services were in the following five most populous countries: Nigeria, the Democratic Republic of the Congo, India, Pakistan, and Ethiopia (the latter for VAS only). The percentage and number of zero-dose children, and the share not receiving VAS and deworming, for each country, region, and globally, are presented in Table 1. The estimate of 19.95 million zero-dose children aged 12–35 months is not directly comparable to the global WHO/UNICEF estimate of 14.5 million zero-dose children, as the latter refers to surviving infants in a single annual birth cohort, whereas our projection covers children aged 12–35 months across the countries included in this analysis.

### 3.3. Zero-Dose Status as an Indicator of Nutritional Vulnerability

We first examined the association between zero-dose status and non-receipt of each nutrition intervention. The correlations were generally weak and positive, with most phi coefficients ranging between 0.1 and 0.3 (Table 2). For VAS, the strongest correlations were observed in Indonesia (φ = 0.35), Chad (φ = 0.33), and Côte d’Ivoire (φ = 0.32), and the weakest in Gabon (φ = 0.06) and India (φ = 0.02). Correlations with non-receipt of deworming followed a similar pattern, with the highest values in Rwanda and Côte d’Ivoire (φ = 0.26) and Chad (φ = 0.25). Across countries and regions, these associations remained below the threshold for a strong correlation. These findings indicate that, while zero-dose children are somewhat less likely to receive nutrition interventions than vaccinated children, zero-dose status alone is a poor predictor of nutrition service access or vulnerability.

**Figure 4:**
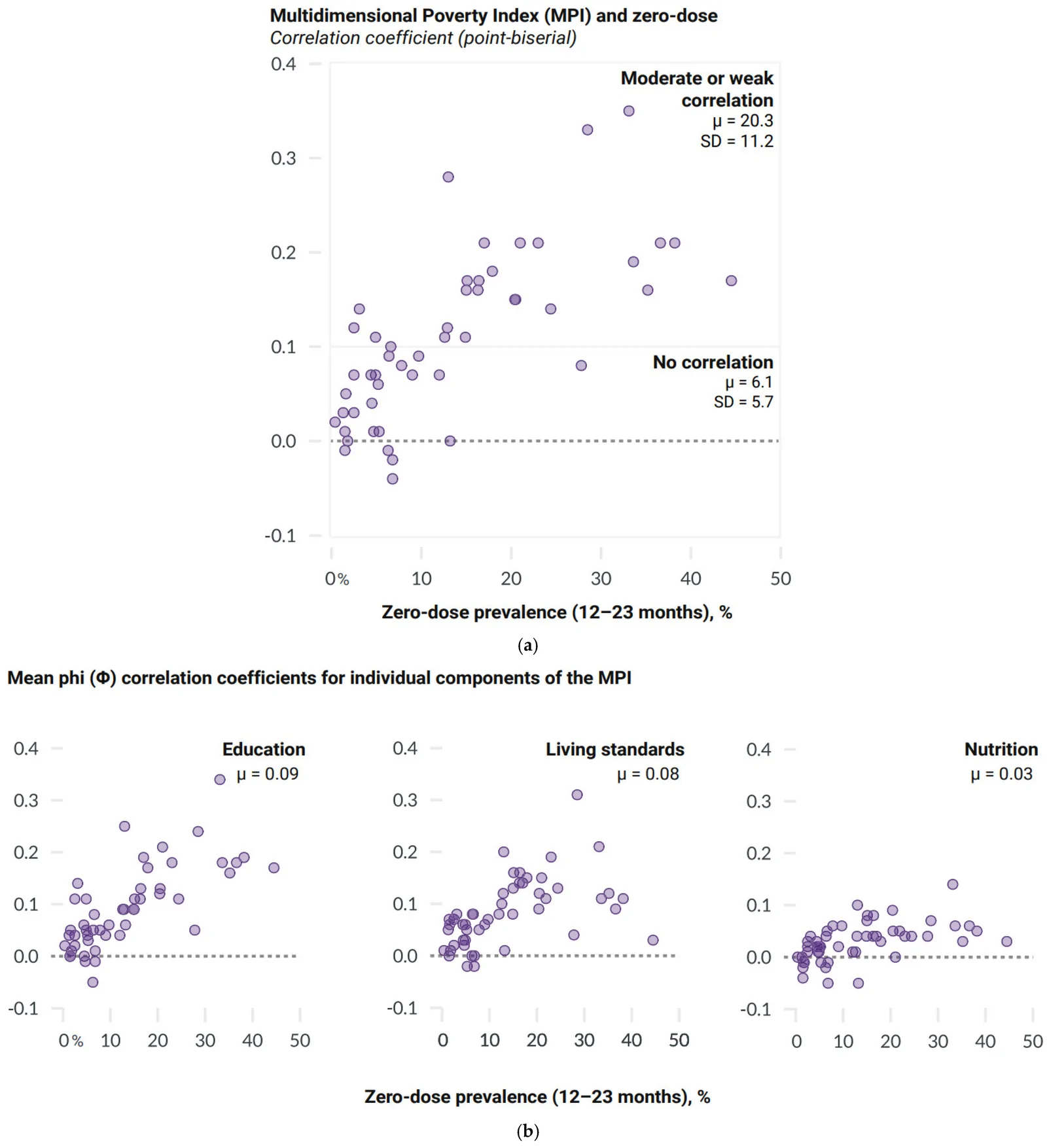
Scatter plot depicting the relationship between national zero-dose prevalence and (**a**) the point-biserial correlation coefficients between zero-dose status and the Multidimensional Poverty Index (MPI) and (**b**) mean phi correlation coefficients for each of the following three MPI dimensions: education, living standards, and nutrition.

We next examined the association between zero-dose status and broader vulnerability, measured by the Multidimensional Poverty Index (Figure 4a). The mean correlation across countries was 0.11, ranging from no correlation (minimum −0.05, São Tomé and Príncipe) to a medium correlation (maximum 0.35, Nigeria). Half of the countries showed no correlation between zero-dose status and the MPI (*n* = 24), while the remainder showed weak (*n* = 24) or medium (*n* = 2) correlations. Among countries with a correlation, the mean zero-dose prevalence was higher (mean 20.3%, SD 11.2) than among countries with no correlation (mean 6.1%, SD 5.7), indicating that the association was strongest where zero-dose children were most prevalent.

Finally, we examined the association between zero-dose status and the individual components of the MPI (Figure 4b). The mean phi coefficient was highest for maternal education (0.09), followed by living standards (0.08), and lowest for child nutritional status (0.03). The association between zero-dose status and the dimension most directly related to nutrition was therefore the weakest of the three.

## 4. Discussion

This study identified a substantial overlap between zero-dose children and non-receipt of critical nutrition interventions, such as high-dose vitamin A supplementation (VAS) and deworming. Our analysis revealed that, globally, three out of every five zero-dose children (or 62%) did not receive VAS, and almost three out of every four (or 72%) did not receive deworming, amounting to an estimated 12.5 and 14.4 million children respectively and concentrated within a small number of high-burden countries. The strength of this overlap, however, was more modest than its scale would suggest. We found that zero-dose status was only weakly correlated with non-receipt of VAS and deworming, with most phi coefficients falling between 0.1 and 0.3, and it was similarly weakly correlated with the Multidimensional Poverty Index (mean of 0.11), likely due to the low coverage of both nutrition services among all children, not just those who were zero-dose. The association between zero-dose status and children’s own nutritional status, as measured by stunting and wasting, was still weaker, with a mean phi coefficient of 0.03. These findings suggest that, although zero-dose children represent a large share of those missing essential nutrition services, zero-dose status does not reliably indicate which children are missing them or are most nutritionally vulnerable, with most correlations falling between 0.1 and 0.3 and the association with child nutritional status approaching zero.

This pattern reflects the generally low coverage of nutrition interventions across all children. In the countries we examined, close to half of all children did not receive VAS (a median of 44%) and more than half did not receive deworming (a median of 53%), regardless of their vaccination status. Zero-dose children were nonetheless more likely not receive than children overall, with mean shares of 60% for VAS and 73% for deworming, compared with these population medians of 44% and 53%. This excess is consistent with the weak positive correlations we observed, but it is modest relative to the high baseline of non-receipt, so that most zero-dose children did not receive these interventions because non-receipt was widespread among all children rather than because zero-dose status specifically marked nutritional risk.

The weak correlations observed across countries indicate that knowing whether a child is zero-dose provides only limited additional information about child VAS or deworming access. This is consistent with prior multi-country analyses showing that coverage of nutrition interventions is generally low and tends to lag the health and immunization platforms through which they could be delivered [41,42]. The weak association between zero-dose status and nutrition service access does not, however, diminish the case for delivering nutrition interventions through immunization platforms. The case rests less on the precision with which zero-dose status identifies nutritional need, as demonstrated by the weak correlations observed across countries, and more on the scale of the opportunity, with an estimated 12.5 million zero-dose children not receiving VAS and 14.4 million not receiving deworming.

Our analysis identified a large absolute number of children were both zero-dose and not receiving nutrition services. By definition, these children have already been missed by routine immunization, and the available evidence indicates that they are characterized by weak and early disengagement from the health system, including home delivery and little or no antenatal care [43]. This has a direct bearing on how nutrition interventions might reach them. Because zero-dose children are precisely those not reached by routine, facility-based services, integration through the routine immunization visit is unlikely to reach them, and the more plausible platform is the outreach and campaign-based delivery used to find and vaccinate zero-dose children, including periodic mechanisms such as Child Health Days [44].

Vitamin A supplementation and deworming are well-suited to this kind of delivery, as they are administered to children of overlapping ages and share many of the logistical requirements of immunization [23]. It is largely through these mechanisms that the two interventions have historically achieved their highest coverage, as in Niger, where integration into national immunization days sustained two-dose VAS coverage above 80% [45], and in South Sudan, where campaign-based delivery raised coverage from 4% to over 76% [46]. The contrast with routine delivery is instructive, in that when Ethiopia shifted VAS from campaigns into the routine health system, coverage fell from above 85% to below 60% and became more unequal across wealth and residence [47]. Delivering nutrition interventions through the platforms that reach zero-dose children could therefore close a substantial access gap, although this benefit is not automatic, as campaign and outreach delivery still tend to under-reach the poorest and most remote children unless they are deliberately targeted [44]. These examples come from other settings and delivery evaluations; our own analysis quantifies the scale of this overlap and the population that could be reached, rather than the effectiveness of integration itself, which would require dedicated evaluation.

These findings have implications for how integration between immunization and nutrition programmes should be designed and targeted. Where the objective is to extend nutrition coverage broadly, campaign-based delivery such as Child Health Days, which reaches children regardless of vaccination status, is the more appropriate channel. The specific value of integrating nutrition into outreach that targets zero-dose children is an equity one: these children are the most excluded and the least likely to be reached by any other means, so bundling nutrition services into the efforts that find them helps reach children who would otherwise be missed entirely. Where the objective is to reach the children who are most nutritionally vulnerable, however, zero-dose status is unlikely to be sufficient on its own and would need to be combined with complementary indicators such as food insecurity, maternal health, and area-level deprivation. This is consistent with evidence that DTP1 alone is an imperfect basis for identifying the communities in which missed children are concentrated and that additional measures of access and deprivation improve targeting [48]. Identifying the most nutritionally vulnerable children therefore requires data and resources beyond zero-dose status alone. The usefulness of zero-dose status as an entry point also appears to vary by context, in that its association with vulnerability was strongest in countries with a high prevalence of zero-dose children. Integration efforts that rely on zero-dose targeting to reach children not receiving nutrition services are therefore likely to be most worthwhile in high-burden settings and of more limited value where zero-dose children are few and dispersed.

This study examined only two nutrition interventions, vitamin A supplementation and deworming. These were selected because they were the interventions for which comparable coverage data were available across surveys, and because they are the nutrition interventions most commonly delivered alongside immunization and most often considered in the integration literature, given their compatibility with vaccination schedules and delivery systems. They represent, however, only part of a broader package of nutrition interventions relevant to integration. Other key interventions, such as caregiver counselling on growth monitoring and the components of infant and young child feeding, including breastfeeding support, the introduction of complementary feeding, and dietary diversity, are essential for addressing child malnutrition [49,50], but could not be examined here because data describing access to these programmes were not available. This gap reflects a wider shortage of data and monitoring for maternal and child nutrition support programmes and limits the extent to which their integration with immunization can currently be assessed. Strengthening the routine measurement of access to this broader package would allow a fuller evaluation of where integration could add the most value.

This study had several limitations. First, the indicators of zero-dose status and of nutrition intervention receipt were derived from caregiver recall and home-based records and are subject to the recognized biases of survey data, including imperfect recall, missing or incomplete vaccination cards, and variation in data collection across countries. Reported receipt of vitamin A supplementation is especially difficult to measure, as there is no standardized method for monitoring its coverage and survey-based estimates depend on caregiver recall, which may affect our estimates of non-receipt [51]. Furthermore, our indicators represent reported non-receipt of interventions rather than confirmed eligibility-based deprivation. Because eligibility for deworming may vary across settings and over time, some children classified as non-recipients may not have been eligible for the intervention under local programme guidance. The DHS indicator records receipt within the previous six months, whereas WHO recommendations may involve annual or biannual preventive chemotherapy depending on local epidemiology. As a result, some children appropriately covered by annual treatment programmes may have been classified as non-recipients, potentially leading to overestimation of programme non-coverage in some settings. Similarly, national eligibility for vitamin A supplementation may have changed over the study period as countries revised guidance in response to improvements in vitamin A status or the introduction of food fortification programmes, and we did not verify whether supplementation remained indicated in each country at the time of its survey. Second, the burden estimates relied on zero-dose and non-receipt prevalences from the year each survey were conducted, which may not reflect current conditions in settings where the public health landscape has since changed. Third, the projected numbers of zero-dose children not receiving these nutrition services were generated using a deterministic model and are presented as point estimates without accompanying measures of uncertainty and should therefore be interpreted as approximate. Fourth, the phi coefficient is mathematically constrained when the two underlying prevalences are far from 50%, as was the case here, so the weak correlations we observed partly reflect the high baseline prevalence of nutrition-service non-receipt rather than the absence of any association. Finally, the Multidimensional Poverty Index, together with stunting and wasting, may not fully capture the complexity of nutritional vulnerability, as these measures lack granularity in reflecting dynamic or context-specific factors.

## 5. Conclusions

Reaching zero-dose children remains one of the central equity goals of global immunization, and the platforms built to find and vaccinate them constitute a substantial and well-supported infrastructure for reaching some of the most excluded children. Our findings indicate that most of these children are also not receiving essential nutrition services, and that the outreach efforts used to reach them therefore offer a practical means of extending essential nutrition interventions to large numbers of children who would otherwise be missed. The value of this opportunity rests, however, on the scale and reach of these platforms rather than on the ability of zero-dose status to identify nutritional need, which our analysis found to be weak. Zero-dose status is better understood as a marker of broad exclusion from the health system than as a specific indicator of nutritional vulnerability, and where the aim is to reach the most nutritionally vulnerable, its use to deliver nutrition services should be accompanied by complementary indicators such as food insecurity and maternal health. Integrating nutrition interventions into platforms that reach zero-dose children represents a potentially valuable opportunity to improve equity in child health, although the effectiveness and operational feasibility of such approaches require dedicated evaluation.

## Figures and Tables

**Table 1:** Percentage (12–23 months) and number (12–35 months) of zero-dose children and the share not receiving vitamin A supplementation (VAS) and preventative chemotherapy for deworming (DW).

	Zero Dose		ZD-No VAS		ZD-No DW	
Geography	%	#	%	#	%	#
**Global**	**-**	**19,950,611**	**62**	**12,457,087**	**72**	**14,403,044**
**East Asia and the Pacific**	**-**	**1,680,286**	**52**	**879,145**	**77**	**1,285,675**
Cambodia	6	46,742	78	36,556	72	33,622
Indonesia	11	849,116	46	390,345	77	651,348
Myanmar	13	214,677	79	169,048	83	177,685
Papua New Guinea	34	155,721	85	132,021	89	139,223
Philippines	12	401,576	36	144,046	69	275,941
Timor-Leste	20	12,454	57	7130	63	7856
**Europe and Central Asia**	**-**	**38,011**	**42**	**15,888**	**88**	**33,514**
Kyrgyz Republic	1	3041	71	2163	99	3033
Tajikistan	7	34,969	39	13,726	87	30,481
**East and Southern Africa**	**-**	**3,536,621**	**74**	**2,625,717**	**33**	**1,163,567**
Burundi	1	10,763	45	4797	47	5025
Comoros	15	7945	61	4847	46	3689
Ethiopia	23	1,969,954	78	1,534,255	-	-
Kenya	3	80,532	52	41,697	61	49,123
Lesotho	1	2124	53	1117	73	1549
Madagascar	21	381,839	79	302,619	72	274,109
Malawi	3	32,862	49	16,070	64	21,108
Mozambique	20	428,545	79	340,499	87	373,598
Namibia	5	7717	25	1918	65	5006
Rwanda	<1%	3837	7	266	5	190
South Africa	6	142,248	67	95,125	80	114,368
Tanzania	5	180,172	72	130,474	74	132,709
Uganda	5	171,808	50	86,146	49	84,736
Zambia	2	17,901	65	11,702	65	11,636
Zimbabwe	10	98,376	55	54,184	88	86,720
**Latin America and Caribbean**	**-**	**99,446**	**84**	**83,611**	**90**	**89,788**
Guatemala	3	13,904	79	10,933	89	12,371
Haiti	16	85,542	85	72,679	91	77,417
**Middle East and North Africa**	**-**	**547,093**	**61**	**336,437**	**91**	**496,051**
Yemen	22	547,093	61	336,437	91	496,051
**South Asia**	**-**	**5,199,511**	**48**	**2,489,905**	**83**	**4,301,920**
Afghanistan	25	746,943	61	453,890	92	662,430
Bangladesh	0	0	-	0	-	0
India	4	2,471,750	54	1,338,507	98	1,948,770
Nepal	5	65,174	20	12,792	46	22,110
Pakistan	13	1,915,645	36	684,717	92	1,668,610
**West and Central Africa**	**-**	**8,849,643**	**68**	**6,026,385**	**79**	**7,032,529**
Benin	15	131,086	60	78,518	78	101,658
Burkina Faso	5	69,267	63	43,943	73	53,592
Cameroon	17	274,618	61	166,820	60	172,113
Chad	38	556,945	74	410,915	83	467,422
Congo	24	69,998	43	29,910	21	15,141
Côte D’Ivoire	28	521,696	61	317,775	61	320,166
Dem. Rep. of the Congo	18	1,377,590	54	740,114	58	800,918
Gabon	15	20,034	77	15,436	34	5464
Gambia	2	3409	72	2470	70	2655
Ghana	3	56,506	46	26,258	74	36,015
Guinea	35	303,365	69	207,830	77	223,029
Liberia	8	25,969	74	19,183	69	17,336
Mali	16	275,896	57	158,450	78	207,636
Mauritania	12	40,836	55	22,516	69	27,661
Niger	13	258,352	58	150,547	82	208,570
Nigeria	33	4,711,341	75	3,525,704	91	4,262,870
Senegal	9	92,842	91	84,546	80	73,064
Sierra Leone	5	22,366	50	11,245	46	9588
Togo	7	37,525	38	14,205	80	27,631

Percentages refer to children aged 12–23 months and (#) numbers to children aged 12–35 months. For the zero-dose columns, values are the prevalence and projected number of zero-dose children. For the ZD–No VAS and ZD–No DW columns, values are the share and projected number of zero-dose children who did not receive the service. A dash indicates a value that was not calculated, either because a pooled percentage was not estimated for regional and global rows, because the survey did not collect the relevant information (deworming in Ethiopia), or because no zero-dose children were identified (Bangladesh).

**Table 2:** Correlation coefficients (ϕ) between zero-dose (ZD) status and non-receipt of VAS and deworming by country.

Geography	Correlation Coefficient (f)		Geography	Correlation Coefficient (f)	
	ZD-VAS	ZD-Deworming		ZD-VAS	ZD-Deworming
**East Asia and the Pacific**			**South Asia**		
Cambodia	0.15	0.16	Afghanistan	0.17	0.10
Indonesia	0.35	0.19	Bangladesh	-	-
Myanmar	0.27	0.19	India	0.02	0.02
Papua New Guinea	0.21	0.18	Nepal	0.29	0.24
Philippines	0.28	0.20	Pakistan	0.16	0.12
Timor-Leste	0.21	0.19	**West and Central Africa**		
**Europe and Central Asia**			Benin	0.16	0.18
Kyrgyz Republic	0.15	0.06	Burkina Faso	0.13	0.13
Tajikistan	0.19	0.07	Cameroon	0.19	0.22
**East and Southern Africa**			Chad	0.33	0.25
Burundi	0.19	0.22	Congo	0.19	0.22
Comoros	0.16	0.11	Côte D’Ivoire	0.32	0.26
Ethiopia	0.16	-	Dem. Rep. of the Congo	0.13	0.15
Kenya	0.21	0.19	Gabon	0.06	0.21
Lesotho	0.22	0.19	Gambia	0.24	0.21
Madagascar	0.22	0.27	Ghana	0.29	0.15
Malawi	0.16	0.13	Guinea	0.16	0.20
Mozambique	0.29	0.25	Liberia	0.17	0.19
Namibia	0.21	0.10	Mali	0.26	0.20
Rwanda	0.30	0.26	Mauritania	0.20	0.19
South Africa	0.18	0.22	Niger	0.23	0.13
Tanzania	0.22	0.19	Nigeria	0.25	0.22
Uganda	0.19	0.20	Senegal	0.10	0.14
Zambia	0.27	0.24	Sierra Leone	0.18	0.18
Zimbabwe	0.22	0.07	Togo	0.25	0.17
**Latin America and Caribbean**					
Guatemala	0.27	0.22			
Haiti	0.27	0.22			
**Middle East and North Africa**					
Yemen	0.24	0.06			

## Data Availability

The data used in this study are publicly available from the Demographic and Health Surveys (DHSs) Programme (https://dhsprogram.com/, accessed on 22 August 2026) and the UNICEF Multiple Indicator Cluster Surveys (MICSs) website (https://mics.unicef.org/, accessed on 22 August 2026). Access to these datasets requires free registration and approval from the respective data providers.

## Abbreviations

DHSs	Demographic and Health Surveys
DTP1	First Dose of the Diphtheria-Tetanus-Pertussis-Containing Vaccine
DW	Deworming
EPI	Expanded Programme on Immunization
Gavi	Gavi, the Vaccine Alliance
IA2030	Immunization Agenda 2030
MICSs	Multiple Indicator Cluster Surveys
MPI	Multidimensional Poverty Index
PHC	Primary Health Care
UNICEF	United Nations Children’s Fund
VAS	Vitamin A Supplementation
WHO	World Health Organization
ZD	Zero-Dose
